# Supplementary material for: The Crunchometer, a low-cost, open-source acoustic analysis of feeding microstructure
Source: eLife. 2026 Jul 14;14:RP108663. doi: 10.7554/eLife.108663 (PMC13368178; doi:10.7554/eLife.108663)
Supplement: Figure 1—source data 1. [file elife-108663-fig1-data1.pdf]

Figure 1-source data 1. Bill of materials

Crunchometer Bill of Materials							
**Main components of Crunchometer**							
**Ítem**	**Description**	**Image**	**Quantity required**	**Unit price USD**	**Webpage seller**	**Model**	**Seller**
**1**	**Condenser Microphone with USB Cable**	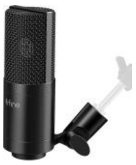	**1**	**$ 63.88**	**https://a.co/d/evaasBZ (Accessed 03-07-2025)**	**K669**	**FIFINE**
**2**	**Global Shutter High Speed 120fps at 1280 x 720p USB Camera with Mini Case with CS mount 2.8-12mm varifocal lens**	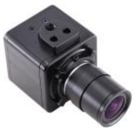	**1**	**$ 107.24**	**https://a.co/d/22F4TgV (Accessed 03-07-2025)**	**KYT-U100-MCS2812GS1**	**Kayeton**
**3**	**Kimble Phenolic Black Screw Cap with Solid PE Liners, Cap Size 22-400 (Case of 144)**	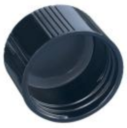	**Bottle cap number depends on the number of pellets measured by the Crunchometer**	**$39.76**	**https://www.amazon.com/-/es/Kimble-fen%C3%B3lico-s%C3%B3lidos-polietileno-paquete/dp/B009EGMU8G?th=1**	**N/A**	**N/A**

					(Accessed 13-11-2025)		
4	UHU Patafix Glue Pad Removable Reusable White	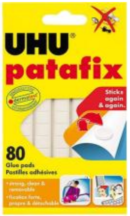	1	~$8-10	https://www.amazon.com/dp/B01DNWS4YK<br><br>(Accessed 25-07-2025)	N/A	N/A
5	Acoustic Panels 12x12x2 Inches Black Pyramid Acoustic Foam	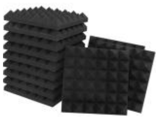	1 package with 12 packs	~$19-20	https://www.amazon.com/dp/B0928PWKH6/ref=sspa_dk_detail_right_aax_0?sp_csd=d2lkZ2V0TmFtZT1zcF9kZXRhaWxfcmlnaHRfc2hhcmVk&th=1<br><br>(Accessed 18-10-2025)	N/A	N/A
6	Acrylic Box	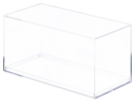	1	N/A	N/A	N/A	N/A
Optional: record liquid consumption (MED-PC required)							

**7**	**Contact lickometer controller with a 28 VDC controlled MED output**	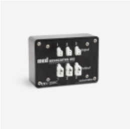	**1**	**~$350 - 400**	**- lickometer-controller (Accessed 3-07-2025)**	**ENV-250C**	**Med Associates inc**
**8**	**ATmega2560 microcontroller board with USB cable.**	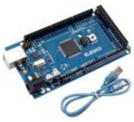	**1**	**~$ 29.4**	**https://a.cod/d/B01H4ZLZLQ (Accessed 3-07-2025)**	**Mega 2560 R3**	**ELEGOO**
