# Supplementary material for: The Crunchometer, a low-cost, open-source acoustic analysis of feeding microstructure
Source: eLife. 2026 Jul 14;14:RP108663. doi: 10.7554/eLife.108663 (PMC13368178; doi:10.7554/eLife.108663)
Supplement: MDAR checklist [file elife-108663-mdarchecklist1.docx]

**Materials Design Analysis Reporting (MDAR)**

**Checklist for Authors**

The [MDAR framework](https://osf.io/xfpn4/) establishes a minimum set of requirements in transparent reporting mainly applicable to studies in the life sciences.

*eLife* asks authors to **provide detailed information within their article** to facilitate the interpretation and replication of their work. Authors can also upload supporting materials to comply with relevant reporting guidelines for health-related research (see [EQUATOR Network](http://www.equator-network.org/%20)), life science research (see the [BioSharing Information Resource](http://biosharing.org/)), or animal research (see the [ARRIVE Guidelines](http://www.plosbiology.org/article/info:doi/10.1371/journal.pbio.1000412) and the [STRANGE Framework](https://doi.org/10.1038/d41586-020-01751-5); for details, see *eLife*’s [Journal Policies](https://reviewer.elifesciences.org/author-guide/journal-policies)). Where applicable, authors should refer to any relevant reporting standards materials in this form.

For all that apply, please note **where in the article** the information is provided. Please note that we also collect information about data availability and ethics in the submission form.

**Materials:**

| **Newly created materials** | **Indicate where provided: section/figure legend** | **N/A** |
| --- | --- | --- |
| The manuscript includes a dedicated "materials availability statement" providing transparent disclosure about availability of newly created materials including details on how materials can be accessed and describing any restrictions on access. |  | N/A |
|  |  |  |
| **Antibodies** | **Indicate where provided: section/figure legend** | **N/A** |
| For commercial reagents, provide supplier name, catalogue number and [RRID](https://scicrunch.org/resources), if available. |  | N/A |
|  |  |  |
| **DNA and RNA sequences** | **Indicate where provided: section/figure legend** | **N/A** |
| Short novel DNA or RNA including primers, probes: Sequences should be included or deposited in a public repository. |  | N/A |
|  |  |  |
| **Cell materials** | **Indicate where provided: section/figure legend** | **N/A** |
| Cell lines: Provide species information, strain. Provide accession number in repository OR supplier name, catalog number, clone number, OR RRID. |  | N/A |
| Primary cultures: Provide species, strain, sex of origin, genetic modification status. |  | N/A |
|  |  |  |
| **Experimental animals** | **Indicate where provided: section/figure legend** | **N/A** |
| Laboratory animals or Model organisms: Provide species, strain, sex, age, genetic modification status. Provide accession number in repository OR supplier name, catalog number, clone number, OR RRID. | This information is provided in Methods:  Vgat-cre mice (JAX:016962 STOCK Slc32a1tm2(cre)Lowl/J)  Vglut2-cre mice ( JAX:016963 STOCK Slc17a6tm2(cre)Lowl/J) |  |
| Animal observed in or captured from the field: Provide species, sex, and age where possible. |  | N/A |
|  |  |  |
| **Plants and microbes** | **Indicate where provided: section/figure legend** | **N/A** |
| Plants: provide species and strain, ecotype and cultivar where relevant, unique accession number if available, and source (including location for collected wild specimens). |  | N/A |
| Microbes: provide species and strain, unique accession number if available, and source. |  | N/A |
|  |  |  |
| **Human research participants** | **Indicate where provided: section/figure legend) or state if these demographics were not collected** | **N/A** |
| If collected and within the bounds of privacy constraints report on age, sex, gender and ethnicity for all study participants. |  | **N/A** |

**Design:**

| **Study protocol** | **Indicate where provided: section/figure legend** | **N/A** |
| --- | --- | --- |
| If the study protocol has been pre-registered, provide DOI. For clinical trials, provide the trial registration number OR cite DOI. |  | N/A |
|  |  |  |
| **Laboratory protocol** | **Indicate where provided: section/figure legend** | **N/A** |
| Provide DOI OR other citation details if detailed step-by-step protocols are available. |  | N/A |
|  |  |  |
| **Experimental study design (statistics details) *** | | |
| **For in vivo studies: State whether and how the following have been done** | **Indicate where provided: section/figure legend. If it could have been done, but was not, write “not done”** | **N/A** |
| Sample size determination | All sample sizes are shown in figure legends | N/A |
| Randomisation | All animals were randomly assigned to experimental groups |  |
| Blinding |  | N/A |
| Inclusion/exclusion criteria | No data was excluded |  |
|  |  |  |
| **Sample definition and in-laboratory replication** | **Indicate where provided: section/figure legend** | **N/A** |
| State number of times the experiment was replicated in the laboratory. |  | N/A |
| Define whether data describe technical or biological replicates. | Biological replicates |  |
|  |  |  |
| **Ethics** | **Indicate where provided: section/submission form** | **N/A** |
| Studies involving human participants: State details of authority granting ethics approval (IRB or equivalent committee(s), provide reference number for approval. |  | N/A |
| Studies involving experimental animals: State details of authority granting ethics approval (IRB or equivalent committee(s), provide reference number for approval. | All experimental procedures were approved by the Cinvestav Animal Care and Use Committee (Cinvestav) under permit number [0080–14] and conducted following the Guide for the Care and Use of Laboratory Animals of the National Institutes of Health. |  |
| Studies involving specimen and field samples: State if relevant permits obtained, provide details of authority approving study; if none were required, explain why. |  | N/A |
|  |  |  |
| **Dual Use Research of Concern (DURC)** | **Indicate where provided: section/submission form** | **N/A** |
| If study is subject to dual use research of concern regulations, state the authority granting approval and reference number for the regulatory approval. |  | N/A |

**Analysis:**

| **Attrition** | **Indicate where provided: section/figure legend** | **N/A** |
| --- | --- | --- |
| Describe whether exclusion criteria were pre-established. Report if sample or data points were omitted from analysis. If yes, report if this was due to attrition or intentional exclusion and provide justification. | No data was excluded from analysis |  |
|  |  |  |
| **Statistics** | **Indicate where provided: section/figure legend** | **N/A** |
| Describe statistical tests used and justify choice of tests. | Data on cumulative feeding time were analyzed using a two sample  Kolmogorov–Smirnov test (two-tailed) to assess differences in distribution.  The one-tailed Mann–Whitney U test for group comparisons, as well as one-way and  two-way ANOVA, were applied, where appropriate, to the crunchometer metrics.  Fisher´s LSD post hoc analysis was performed. α was always set p < 0.05.  To quantify the relationship between neuronal activity and ingestive behaviors, we  used receiver operating characteristic (ROC) analysis to test whether calcium signals  could predict feeding or licking events. |  |
|  |  |  |
| **Data availability** | **Indicate where provided: section/submission form** | **N/A** |
| For newly created and reused datasets, the manuscript includes a data availability statement that provides details for access (or notes restrictions on access). | Benchmark and Crunchometer software will be available on OSF (Arroyo et al., 2025)  using the following link:  <https://osf.io/bmkdc/?view_only=913feef710714d2bbd4cfb45960fb7be> |  |
| When newly created datasets are publicly available, provide accession number in repository OR DOI and licensing details where available. | Arroyo B, Pérez-Ortega J, Lopez A, Hernandez-Lemus E, Gutierrez R. 2025. Software and  Benchmark: The Crunchometer: A Low-Cost, Open-Source Acoustic Analysis of  Feeding Microstructure. DOI: <https://doi.org/10.17605/OSF.IO/BMKDC> |  |
| If reused data is publicly available provide accession number in repository OR DOI, OR URL, OR citation. |  | N/A |
|  |  |  |
| **Code availability** | **Indicate where provided: section/figure legend** | **N/A** |
| For any computer code/software/mathematical algorithms essential for replicating the main findings of the study, whether newly generated or re-used, the manuscript includes a data availability statement that provides details for access or notes restrictions. | Matlab 2026a |  |
| Where newly generated code is publicly available, provide accession number in repository, OR DOI OR URL and licensing details where available. State any restrictions on code availability or accessibility. | Benchmark and Crunchometer software will be available on OSF (Arroyo et al., 2025) using the following link:  <https://osf.io/bmkdc/?view_only=913feef710714d2bbd4cfb45960fb7be>  The OSF repository contains two folders under **Files**:  **TheCrunchometerV2_Matlab** includes both the classic scripts and an App GUI version, compatible with Windows, macOS (Intel and Apple Silicon), and Linux. This version requires a MATLAB license and FFmpeg and is limited to videos up to 4 hours long.  The **TheCrunchometerV2_Python** folder contains a standalone macOS Silicon executable (TheCrunchometer-2.0.0-macOS-arm64.dmg); this is the recommended version. It requires no MATLAB license or additional dependencies, supports 24-hour video files, and runs optimally on Apple Silicon hardware (e.g., Mac mini M4). To install, double-click the .dmg file and authorize the application via Apple menu → System Settings → Privacy & Security → Open Anyway.  A standalone Windows executable is also available; it handles 24-hour videos but runs more slowly than the macOS build.  Alternatively, a Python source version of the Crunchometer is available on GitHub:<https://github.com/RanierLabNeurobiologyAppetite2026/TheCrunchometerPy>  We recommend directly downloading the standalone software from GitHub using the following link:  MacOS Silicon: [**TheCrunchometer-2.0.0-macOS-arm64.dmg**](https://github.com/RanierLabNeurobiologyAppetite2026/TheCrunchometerPy/releases/download/v2.0.0/TheCrunchometer-2.0.0-macOS-arm64.dmg)  To install, double-click the .dmg file and authorize the application via Apple menu → System Settings → Privacy & Security → Open Anyway.  Windows: [**Setup_TheCrunchometer-2.0.0-Windows-x64.exe**](https://github.com/RanierLabNeurobiologyAppetite2026/TheCrunchometerPy/releases/download/v2.1.0/Setup_TheCrunchometer-2.0.0-Windows-x64.exe)  An example folder, CrunchometerRunExample, is also provided, containing Audio_Data (.ogg) and Video_Data (.mkv) subfolders from a 10-minute recording for testing the Crunchometer software.  A tutorial video on installing and running the Crunchometer is available at the following [link](https://youtu.be/cubBnfOoGy8). |  |
| If reused code is publicly available provide accession number in repository OR DOI OR URL, OR citation. |  | N/A |

**Reporting:**

The MDAR framework recommends adoption of discipline-specific guidelines, established and endorsed through community initiatives.

| **Adherence to community standards** | **Indicate where provided: section/figure legend** | **N/A** |
| --- | --- | --- |
| State if relevant guidelines (e.g., ICMJE, MIBBI, ARRIVE, STRANGE) have been followed, and whether a checklist (e.g., CONSORT, PRISMA, ARRIVE) is provided with the manuscript. |  | N/A |

* We provide the following guidance regarding transparent reporting and statistics; we also refer authors to [Ten common statistical mistakes to watch out for when writing or reviewing a manuscript](https://doi.org/10.7554/eLife.48175).

**Sample-size estimation**

- You should state whether an appropriate sample size was computed when the study was being designed
- You should state the statistical method of sample size computation and any required assumptions
- If no explicit power analysis was used, you should describe how you decided what sample (replicate) size (number) to use

**Replicates**

- You should report how often each experiment was performed
- You should include a definition of biological versus technical replication
- The data obtained should be provided and sufficient information should be provided to indicate the number of independent biological and/or technical replicates
- If you encountered any outliers, you should describe how these were handled
- Criteria for exclusion/inclusion of data should be clearly stated
- High-throughput sequence data should be uploaded before submission, with a private link for reviewers provided (these are available from both GEO and ArrayExpress)

**Statistical reporting**

- Statistical analysis methods should be described and justified
- Raw data should be presented in figures whenever informative to do so (typically when N per group is less than 10)
- For each experiment, you should identify the statistical tests used, exact values of N, definitions of center, methods of multiple test correction, and dispersion and precision measures (e.g., mean, median, SD, SEM, confidence intervals; and, for the major substantive results, a measure of effect size (e.g., Pearson's r, Cohen's d)
- Report exact p-values wherever possible alongside the summary statistics and 95% confidence intervals. These should be reported for all key questions and not only when the p-value is less than 0.05.

**Group allocation**

- Indicate how samples were allocated into experimental groups (in the case of clinical studies, please specify allocation to treatment method); if randomization was used, please also state if restricted randomization was applied
- Indicate if masking was used during group allocation, data collection and/or data analysis
