# Supplementary material for: The Crunchometer, a low-cost, open-source acoustic analysis of feeding microstructure
Source: eLife. 2026 Jul 14;14:RP108663. doi: 10.7554/eLife.108663 (PMC13368178; doi:10.7554/eLife.108663)
Supplement: Peer review history

# Editor report: eLife Assessment

**DOI:** 10.7554/eLife.108663.3.sa0

**Authors:** Yunlei Yang

**Affiliations:** Albert Einstein College of Medicine United States

**Keywords:** Convincing, Important

This **important** manuscript presents the Crunchometer, an open-source and low-cost acoustic system for high-resolution quantification of biting and chewing in mice. The work addresses a need for reliable measures of food consumption and feeding microstructure, and the tool has broad relevance for studies of ingestive behavior, appetite circuits, hypothalamic function, and pharmacological interventions. The evidence supporting the methodological advance is **convincing**, and the Crunchometer outputs were carefully validated against human observer scoring, reliably distinguished biting and chewing events, and captured changes in feeding behavior across different foods, physiological states, and semaglutide treatment. The study also demonstrates that the system can reveal biologically meaningful features of feeding, including meal structure, bite and chew dynamics, and altered consumption patterns after pharmacological manipulation. A significant additional contribution is the identification of previously unrecognized meal-related neurons in the lateral hypothalamus, providing novel circuit-level insight into solid food consumption and naturalistic feeding behavior. Although some neuroscience conclusions remain more preliminary than the methodological validation, the study provides strong evidence for the utility of the Crunchometer and will be of interest to researchers studying ingestive behavior, hypothalamic circuits, and metabolic regulation.

# Referee report: Reviewer #1 (Public review)

**DOI:** 10.7554/eLife.108663.3.sa1

This is an interesting and valuable paper by Gil-Lievana, Arroyo et al. that presents an open-source method (the "Crunchometer") for quantifying biting and chewing behavior in mice using audio detection. The work addresses an important and unmet need in the field: quantitative measures of feeding behavior with solid foods, since most prior approaches have been limited to liquids. The authors make a clear and compelling case for why this problem is important, and I fully agree with their motivation.

The system is carefully validated against human-scored video data and is shown to be at least as accurate, and in some cases more accurate, than human observers. This is a major strength of the study. I also particularly appreciate the demonstration of the technology in the context of LHA circuitry, which nicely illustrates its utility and importance for mechanistic studies of feeding. I also appreciate the ability to readily time lock neural data to individual crunches. Overall, the manuscript is well executed and represents a useful contribution to the field.

Comments on revised version.

The revised manuscript has addressed my minor initial concerns. I appreciate that the sample size was increased for the recording experiments.

# Referee report: Reviewer #2 (Public review)

**DOI:** 10.7554/eLife.108663.3.sa2

Summary:

The authors set out to develop and validate the Crunchometer, a low-cost, open-source acoustic system designed to overcome the limitations of existing methods for studying feeding behavior in rodents. Their goal was to provide a tool that could precisely capture the microstructure of solid food intake, something often overlooked in favor of liquid-based assays, while being affordable, scalable, and compatible with neural recording techniques. By doing so, they aimed to enable detailed analysis of how physiological states, drugs, and specific neural circuits shape naturalistic feeding behaviors.

Strengths:

(1) Introduces a low-cost, open-source acoustic tool for measuring solid food intake, filling a critical gap left by expensive and proprietary systems.

(2) Makes the method easily adoptable across labs with detailed setup instructions and shared benchmark datasets.

(3) Provides high temporal precision for detecting bite events compared to human observers.

(4) Successfully distinguishes feeding microstructure (bites, bouts, IBIs, gnawing vs. consumption) with greater objectivity than manual annotation.

(5) Demonstrates compatibility with electrophysiology and calcium imaging, enabling fine-scale alignment of neural activity with feeding behavior.

(6) Effectively discriminates between fed vs. fasted states, validating physiological sensitivity.

(7) Captures pharmacological effects of semaglutide, although this is really just reduced feeding and associated readouts (bouts, latency, etc.)

(8) Has potential to distinguish consummatory vs. non-consummatory behaviors (e.g., food spillage, gnawing), however the current SVM model struggles to separate biting from gnawing due to similar acoustic profiles and manual validation is still required.

(9) Provides potential for closed-loop experiments

Weaknesses:

(1) Some neuroscience findings (calcium imaging of GABAergic vs. glutamatergic neurons) are based on small pilot samples (n=2 mice per condition), limiting generalizability.

(2) Chemogenetic and pharmacological experiments used small cohorts, raising statistical power concerns.

(3) Correlation with actual food intake is modest and sometimes less accurate than human observers

(4) Sensitive to hoarding behavior, which can reduce detection accuracy and requires manual correction for misclassifications (e.g., tail movements, non-food noises). However, these limitations are discussed and not ignored.

Comments on revised version.

The authors have addressed all my comments and have put forth a creative, accurate approach to assessing food intake in rodents.

# Author comment: Author response

**DOI:** 10.7554/eLife.108663.3.sa3

**Authors:** Elvi Gil Lievana, Benjamin Arroyo, Jesús Pérez-Ortega, Axel Lopez, Luis Rodriguez-Blanco, Xarenny Diaz, Gustavo Hernandez, Alam Coss, Emily Alway, Naama Reicher, Enrique Hernández-Lemus, Maya Kaelberer, Diego V Bohórquez, Ranier Gutierrez

**Affiliations:** Laboratory of Neurobiology of Appetite, Department of Pharmacology, CINVESTAV Mexico City Mexico; Laboratory of Neurobiology of Appetite, Department of Pharmacology, CINVESTAV Mexico City Mexico; Center for Research and Advanced Studies of the National Polytechnic Institute Mexico City Mexico; Laboratory of Neurobiology of Appetite, Department of Pharmacology, CINVESTAV Mexico city Mexico; Laboratory of Neurobiology of Appetite, Department of Pharmacology, CINVESTAV Mexico City Mexico; Laboratory of Neurobiology of Appetite, Department of Pharmacology, CINVESTAV Mexico City Mexico; Laboratory of Neurobiology of Appetite, Department of Pharmacology, CINVESTAV; Laboratory Neurobiology of Appetite, Center for Research on Aging (CIE), CINVESTAV, Sede Sur, Mexico City, Mexico Mexico City Mexico; Laboratory of Neurobiology of Appetite, Department of Pharmacology, CINVESTAV; Laboratory Neurobiology of Appetite, Center for Research on Aging (CIE), CINVESTAV, Sede Sur, Mexico City, Mexico Mexico City Mexico; Laboratory of Gut-Brain Neurobiology, Duke University, Durham, NC 27710; USA; Department of Neurobiology, Duke University, Durham, NC 27710; USA; Department of Medicine, Duke University, Durham, NC 27710; USA Durham, North Carolina United States; Laboratory of Gut-Brain Neurobiology, Duke University, Durham, NC 27710; USA; Department of Neurobiology, Duke University, Durham, NC 27710; USA; Department of Medicine, Duke University, Durham, NC 27710; USA Durham, North Carolina United States; Computational Genomics Division, National Institute of Genomic Medicine (INMEGEN), Mexico City, Mexico; Center for Complexity Sciences, Universidad Nacional Autónoma de México, Mexico City, Mexico Ciudad de México Mexico; Department of Medicine, Duke University, Durham, NC 27710, USA; Department of Physiology, University of Arizona, Tucson, AZ 85721, USA Tucson, Arizona United States; Duke University Durham United States; Laboratory Neurobiology of Appetite, Department of Pharmacology, CINVESTAV, 07360, Mexico City, Mexico; Laboratory Neurobiology of Appetite, Center for Research on Aging (CIE), CINVESTAV, Sede Sur, Mexico City, Mexico Mexico City Mexico

The following is the authors’ response to the original reviews.

**eLife Assessment**

This valuable manuscript presents an open-source and low-cost acoustic system for quantifying biting and chewing in mice. The approach is carefully validated against human observers, demonstrating strong methodological reliability and enabling high-resolution analysis of feeding microstructure. The tool has broad relevance for studies of appetite circuits and pharmacological interventions. An important contribution is the identification of previously unrecognized "meal-related" neurons in the lateral hypothalamus, providing novel biological insight into solid food consumption. While the support for the methodological advances is compelling and robust, some circuit-level conclusions are preliminary or incomplete, relying on small pilot samples and manual classification, and should be interpreted with caution. This paper will be of interest to those interested in ingestive behavior and/or the hypothalamus.

We thank the reviewers for their careful reading and constructive comments, which have substantially strengthened the manuscript. In the revised version, we have addressed every suggestion and introduced the following major additions: New experiments. We added one additional Vglut2 mouse to the calcium imaging cohort, achieving 386 neurons (Figure 8), and three naive Vgat mice with unilateral DREADD injections (Supplementary Fig. 5-1). New analyses. We performed ROC analyses on all feeding- and licking-related responses of n = 79 LH GABAergic and n = 386 LH glutamatergic neurons (Figures 7D-F and 8D-F). We also characterized the robustness of the Crunchometer to additive white-noise injection (Supplementary Fig. 1-2). New supplementary material. Three new supplementary figures have been added in total (Supplementary Figs. 1-2, 5-1, and 6-1). Supplementary Fig. 6-1 provides instructions for building a 1-Hz pulse generator that blinks an LED in synchrony with the video. Software improvements. We upgraded the original MATLAB scripts to an App GUI version, migrated the full codebase from MATLAB to Python, and packaged it as fully standalone executables for macOS (Apple Silicon) and Windows both of which run without a MATLAB license.

Our point-by-point responses to the reviewers' comments are in red below. Deletions are omitted for brevity. We hope that the revisions fully address the points raised and render the manuscript suitable for publication.

**Public Reviews:**

**Reviewer #1 (Public review):**

This is an interesting and valuable paper by Gil-Lievana, Arroyo et al. that presents an open-source method (the "Crunchometer") for quantifying biting and chewing behavior in mice using audio detection. The work addresses an important and unmet need in the field: quantitative measures of feeding behavior with solid foods, since most prior approaches have been limited to liquids. The authors make a clear and compelling case for why this problem is important, and I fully agree with their motivation.

The system is carefully validated against human-scored video data and is shown to be at least as accurate, and in some cases more accurate, than human observers. This is a major strength of the study. I also particularly appreciate the demonstration of the technology in the context of LHA circuitry, which nicely illustrates its utility and importance for mechanistic studies of feeding. I also appreciate the ability to readily time-lock neural data to individual crunches. Overall, the manuscript is well-executed and represents a useful contribution to the field.

We thank you for your appreciation of the Crunchometer and its alignment with ephys:

To further facilitate alignment with neuronal activity, we have now also included a schematic diagram of the pulse generator used to blink an LED in synchronization with the video (see the new Supplementary Fig. 6-1).

The comments I have are largely minor and should be straightforward to address:

(1) The authors should report sample sizes for all mouse cohorts, either alongside the statistics or in the figure legends for mean data.

We apologize for this oversight. We have now included all sample sizes in the figure captions.

(2) Clarification is needed as to whether crunch detection fidelity is influenced by the hardness or softness of the food. The focus here is on standard pellets, with some additional high-fat pellet data, but it would be useful to know how generalizable the method is across different textures.

We thank the reviewer for this important observation. Because the Crunchometer depends on bites generating an audible acoustic signal, food hardness directly impacts detection fidelity. Hard, brittle foods are readily detected, whereas soft foods such as jelly, pudding, or peanut butter are unlikely to produce a reliably detectable signal. This is a genuine scope limitation of the method, and we now make it explicit in the manuscript (see below).

Regarding the two diets used in our study, Chow and HFD pellets differ only slightly in consistency, with HFD being marginally softer. These differences proved too subtle to separate acoustically: the intensity (dB) and spectral content of bites on the two diets were closely overlapping. Accordingly, when we trained an SVM on audio features alone, it could not reliably discriminate Chow from HFD bites.

Importantly, the Crunchometer does not need to resolve food identity from sound, because audio and video play complementary roles in the system: the acoustic channel confirms that a bite occurred, while the mouse's position within the food-specific ROI determines which food was consumed. This division of labor is what allows per-diet attribution despite acoustically similar pellets.

We have added to the Result section:

“The Crunchometer, therefore, does not need to infer food identity acoustically: audio confirms that a bite occurred, and the mouse's position within a food-specific ROI identifies which food was consumed. This design enables per-diet attribution even for pellets with indistinguishable crunch signatures.”

We fully agree with the reviewer that the study of solid-food consumption should not be restricted to standard murine diets. Foods with naturalistic textures, for example, the Granny Smith apple, chocolate, and salted peanuts used by O'Connell et al. (2025), span a much wider range of hardness and elasticity than Chow vs. HFD, and would likely generate more clearly differentiated acoustic signatures. We hypothesize that the Crunchometer could generalize to such foods to the extent that each food produces a clear and distinct acoustic pattern, and even where acoustic signatures overlap, ROI-based spatial attribution would continue to resolve food identity as long as each food is presented at a separate, trackable location.

To make this scope explicit for readers, we have added the following clarification to the Behavioral Protocol section:

"Our study is limited to the acoustic detection of standard Chow and HFD pellets, both of which exhibit a firm, brittle consistency. Future work should evaluate the fidelity of the Crunchometer across a broader range of food textures, encompassing varying degrees of hardness and elasticity, as explored by O'Connell et al. (2025)."

(3) The authors should comment on how susceptible the Crunchometer is to background noise. For example, how well does it perform in the presence of white noise, experimenter movement, or other task-related sounds?

We thank the reviewer for this valuable comment. The Crunchometer performs reliably in controlled, low-noise environments, but like any acoustic detection system, it is vulnerable to interference from sounds whose spectral content overlaps with the bite-related frequency band (500–950 Hz). To quantify this vulnerability, we stress-tested both the threshold-based and SVM-based detection methods by adding white noise to the original audio recordings at progressively decreasing amplitudes and measuring how detection performance degraded as the signal-to-noise ratio decreased. We found that the threshold-based method was more robust to white-noise contamination than the SVM-based method, maintaining acceptable detection performance at lower SNR values before degrading [see the new Supplementary Fig. 1-2].

First, the white noise amplitude is generated as follows:

$$\displaystyle A_{\text {noiss }}=10^{\left(L_{\text {solis }} / 20\right)}$$

Where *L*_𝑛𝑜𝑖𝑠𝑒_ is the desired amplitude of the White Noise in dB. Then, the audio signal was range-normalized to its absolute maximum value, and the white noise was added with its desired amplitude, as shown by the following formula:

$$\displaystyle n(t)=\operatorname{signal}+\left(A_{\text {noise }} * \operatorname{randn}(\operatorname{size}(\operatorname{signal}))\right)$$

(4) Chemogenetic activation of LHA GABAergic neurons is used. DREADD-based activation may strongly drive these neurons in a way that is not directly comparable to optogenetic or more physiological manipulations. While I do not think additional experiments are required, it would strengthen the discussion to briefly acknowledge this limitation.

We thank the Reviewer for this thoughtful observation, which we agree with. Chemogenetic activation of LHA GABAergic neurons via DREADDs does not reproduce the physiological firing dynamics of these neurons along several dimensions: it imposes a sustained, tonic drive lasting hours after CNO administration; it likely produces firing rates above the endogenous range; and it lacks the fine temporal structure, phasic bursts, behaviorally- phased locked activity that these neurons exhibit during natural feeding episodes.

We recognize, however, that this limitation is not unique to chemogenetics. Optogenetic approaches likewise fail to reproduce endogenous activity, as they impose synchronous, high-frequency activation patterns on a single cell type that are unlikely to occur under physiological conditions. Moreover, as we previously described in a phenomenon our laboratory termed *optoception* (Luis-Islas et al., 2022), optogenetic stimulation can itself generate signals perceptible to the animal, adding a further interpretive caveat. Thus, both techniques depart from physiological activity.

For these reasons, we interpret our findings as evidence that activation of LHA GABAergic neurons is sufficient to drive the observed behavioral effects, without claiming that the endogenous firing pattern encodes these behaviors in the same manner or with the same dynamics imposed by our manipulation. We have now added a brief statement to the Discussion acknowledging this limitation explicitly:

“A methodological consideration is that chemogenetic activation via DREADDs imposes a sustained, supra-physiological drive that does not reproduce the temporal structure of endogenous LHA GABAergic activity during feeding; optogenetic manipulations share analogous limitations (see optoception; Luis-Islas et al., 2022). Our findings, therefore, establish that activation of this neuronal population is sufficient to produce uncontrolled feeding and gnawing, without implying that its endogenous firing encodes them in the same manner.”

**Reviewer #2 (Public review):**

Summary:

This manuscript introduces the Crunchometer, a low-cost, open-source acoustic platform for monitoring the microstructure of solid food intake in mice. The Crunchometer is designed to overcome the limitations of existing methods for studying feeding behavior in rodents. The goal was to provide a tool that could precisely capture the microstructure of solid food intake, something often overlooked in favor of liquid-based assays, while being affordable, scalable, and compatible with neural recording techniques. By doing so, the authors aimed to enable detailed analysis of how physiological states, drugs, and specific neural circuits shape naturalistic feeding behaviors.

Strengths:

The study's strengths lie in its clear innovation, methodological rigor in validation against human annotation, and demonstration of broad utility across behavioral and neuroscience paradigms. The approach addresses a significant methodological gap in the field by moving beyond liquid-based feeding assays and provides an accessible tool for precisely dissecting ingestive behavior. The system is validated across multiple contexts, including physiological state (fed vs. fasted), pharmacological manipulation (semaglutide), and circuit-level interventions (chemogenetic activation of LH neurons), and is further shown to integrate seamlessly with both electrophysiology and calcium imaging.

(1) Introduces a low-cost, open-source acoustic tool for measuring solid food intake, filling a critical gap left by expensive and proprietary systems.

(2) Makes the method easily adoptable across labs with detailed setup instructions and shared benchmark datasets.

(3) Provides high temporal precision for detecting bite events compared to human observers.

(4) Successfully distinguishes feeding microstructure (bites, bouts, IBIs, gnawing vs. consumption) with greater objectivity than manual annotation.

(5) Demonstrates compatibility with electrophysiology and calcium imaging, enabling fine-scale alignment of neural activity with feeding behavior.

(6) Effectively discriminates between fed vs. fasted states, validating physiological sensitivity.

(7) Captures the pharmacological effects of semaglutide, although this is really just reduced feeding and associated readouts (bouts, latency, etc).

(8) Has potential to distinguish consummatory vs. non-consummatory behaviors (e.g., food spillage, gnawing); however, the current SVM model struggles to separate biting from gnawing due to similar acoustic profiles, and manual validation is still required.

(9) Provides potential for closed-loop experiments.

Weaknesses:

Several limitations temper the strength of the conclusions: the supervised classifier still requires manual correction for gnawing, generalizability across different setups is limited, and the neuroscience findings, particularly calcium imaging of GABAergic and glutamatergic neurons, are based on small pilot samples. These issues do not undermine the value of the tool, but mean that the neural circuit findings should be interpreted as preliminary.

We sincerely thank the Reviewer for the careful and generous reading of our manuscript, and particularly for recognizing the methodological gap that the Crunchometer seeks to fill. We appreciate the acknowledgment that the tool's validation spans physiological, pharmacological, and circuit-level contexts, and that its integration with electrophysiology and calcium imaging was considered seamless. The Reviewer has also accurately identified the three main limitations of the current version of the platform, which we address in turn below:

(1) The supervised SVM classifier still requires manual correction for gnawing.

We agree with the Reviewer. The acoustic signatures of biting (consummatory) and gnawing (non-consummatory manipulation of the pellet) share overlapping linear spectrotemporal features that our SVM exploits for discrimination. This overlap reflects a genuine biomechanical similarity (both involve incisor contact with the pellet surface) rather than a shortcoming of the classifier per se. In ongoing work toward Crunchometer 2.0, we are addressing these limitations. The Crunchometer 2.0 will incorporate more sophisticated deep learning algorithms, such as ResNet, to better exploit non-linear features. Also, we are currently collecting a larger database of bite, gnawing, and environmental noise sounds across different setups, microphones, and conditions to build a more robust dataset for training new AI algorithms that can discriminate between gnawing and biting and generalize more robustly across microphones and behavioral setups. This effort will also be important for developing a closed-loop version of the Crunchometer to detect bites in real time and trigger an actuator (e.g., a laser). But we agree that, for the present manuscript, gnawing classification remains the weakest link in the pipeline.

Nevertheless, we think that having a human in the loop is an advantage (not a disadvantage) of the equipment, as it improves the quality of database curation. No matter how sophisticated future algorithms become, human intervention will remain essential. To this end, we have now developed a human-validation GUI that further facilitates human revision of snippets through an intuitive, easy workflow, reducing human effort (Author response image 1).

**Author response image 1.** The visual validator GUI allows a human to verify and reclassify snippets into the correct category in a friendly interface.

(1) Generalizability across different setups is limited.

This is a fair concern and one we have taken seriously, as noted above, and one we have already recognized. The acoustic signal captured by the Crunchometer is inherently sensitive to the geometry and material of the box, microphone placement, the ambient noise floor of the vivarium or experimental room, and the hardness of the specific pellet batch. To mitigate this, we have (1) released the full hardware specifications and bill of materials so that other laboratories can reproduce the acquisition geometry, and (2) provided the benchmark dataset and trained classifier weights so that groups using comparable setups can deploy the tool directly. We have already acknowledged that the SVM does not always generalize across setups. In this regard, we have now shown that the threshold method is more resistant to white-noise contamination (see new Supplementary Fig. 1–2) and, in our experience in the lab, it performs robustly across multiple setups and conditions we have tested. More importantly, improved algorithms are currently under development in our laboratory.

(1) Some neuroscience findings (calcium imaging of GABAergic vs. glutamatergic neurons) are based on small pilot samples (n=2 mice per condition), limiting generalizability.

(3) The neuroscience findings (calcium imaging of GABAergic and glutamatergic LH neurons) are based on small pilot samples.

The Reviewer is correct, and we appreciate the comment. As noted in the manuscript, we explicitly state in the Results and Discussion that these findings are presented as preliminary. As the Reviewer noted, these findings do not undermine the value of the Crunchometer; we fully agree. The calcium imaging experiments were designed as a proof-of-concept to demonstrate that the temporal precision of the Crunchometer is sufficient to align neural activity with individual bite events, rather than as a definitive circuit-level characterization of LH GABAergic and glutamatergic populations during feeding. Nevertheless, we have now increased the number of Vglut2 mice by 1, bringing the total number of glutamatergic neurons to 386. We have now also performed a formal quantification of all the experiments recorded in Vgat (n=2, three sessions, 79 neurons) and Vglut2 (n=3, 6 sessions, 386 neurons). This new formal analysis uncovers neurons selectively tuned to liquid, solid, and both food types. A fully powered characterization of these two populations is underway in our laboratory, once funding arrives in the lab, and will be reported in a dedicated follow-up study.

(2) Chemogenetic and pharmacological experiments used small cohorts, raising statistical power concerns.

The chemogenetic experiments were conducted with a modest sample size (n = 4 bilaterally infected mice). Nevertheless, the data revealed a robust, reproducible behavioral effect consistent across all four subjects. The primary aim of this study was to illustrate the potential utility of the Crunchometer using complementary experimental approaches, including chemogenetic activation of GABAergic neurons in the lateral hypothalamic area (LHA). To further address this concern, we have now included three additional transgenic mice with unilateral infections and obtained results comparable to those of the bilateral condition. These new data are presented in a new supplementary figure comparing unilateral and bilateral infections (Supplementary Fig. 5-1). Notably, chemogenetic activation of LHA GABAergic neurons promoted eating-related consummatory behaviors to a similar extent under both unilateral and bilateral DREADD activation. Accordingly, we have now added the following text to the Results section:

“Notably, unilateral DREADD infections in other naïve n=3 Vgat-cre mice yielded results comparable to bilateral infections. While the effect size was slightly reduced with unilateral administration, the difference between the two delivery methods was not statistically significant (Supplementary Fig. 5-1)”

(3) Correlation with actual food intake is modest and sometimes less accurate than human observers.

We agree that this result highlights the complexity of feeding behavior, influenced by factors such as hoarding and spillage. The threshold method detects feeding behavior solely based on the magnitude of bite-related sounds (e.g., when the mouse bites the pellet close to the microphone), whereas human observers incorporate additional visual information to infer feeding behavior even in the absence of detectable chewing sounds, introducing variability in detection criteria. Although the number of bouts identified by the Threshold method was comparable to those annotated by human observers, the estimated duration (Bout Size) of those detections differed. This discrepancy likely reflects some inconsistency in the detection criteria among human observers and delays in identifying the onset. Moreover, instances of mice chewing pellets without consuming them (i.e., spillage) were observed. These events were often misclassified as feeding bouts, resulting in false positives for both the threshold method and human observers.

(4) Sensitive to hoarding behavior, which can reduce detection accuracy and requires manual correction for misclassifications (e.g., tail movements, non-food noises). However, these limitations are discussed and not ignored.

We thank the reviewer for this constructive comment and for acknowledging that we explicitly discuss these limitations rather than overlook them. Indeed, gnawing and hoarding behaviors (together with tail movements and non-food noises) are factors that can reduce the accuracy of feeding detection. Even using the Crunchometer, an accurate measurement of solid-food consumption therefore remains challenging, which further supports the inclusion of a human-in-the-loop step to ensure a high-quality, well-curated database. Accordingly, we have added the following sentence to the Result section:

"This human validation was essential for ensuring the high fidelity of our behavioral database and mitigating the inherent limitations of automated classification."

Conclusion:

Overall, this is an exciting and impactful methodological advance that will likely be widely adopted in the field. I recommend minor revisions to clarify the limits of classifier generalizability, better contextualize the small-sample neuroscience findings as pilot data, and discuss future directions (e.g., real-time closed-loop applications).

We thank you for your constructive comments.

**Reviewer #3 (Public review):**

Summary:

The manuscript provides detailed information on the construction of open-source systems to monitor ingestive behavior with low-cost equipment. Overall, this is a welcome addition to the arsenal of equipment that could be used to make measurements. The authors show interesting applications with data that reveal important neurophysiological properties of neurons in the lateral hypothalamus. The identification of previously unknown "meal-related" neurons in the LH highlights the utility of the device and is a novel insight that should spark further investigation on the LH. This manuscript and videos provide a wealth of useful information that should be a must-read for anyone in the ingestive behavior or hypothalamus fields.

A scholarly introduction to the history and utility of various ways feeding is measured in rodents is provided. One point - the microstructure of eating solid food - has been studied extensively (for one of many studies, see https://doi.org/10.1371/journal.pone.0246569). However, I agree that the crunchometer will allow for more people to access recordings during food intake and temporally lock consummatory behavior to neural activity.

Apologize for this oversight. This is indeed an important reference for the microstructure of eating solid food in a social context. We have now included it in the Introduction of this reference “Food intake in social contexts is a more ethologically valid model, in which radio-frequency identification (RFID) transponders enable the simultaneous assessment of feeding behavior across multiple mice in a single box (Rathod and Fulvio, 2021)”

Questions on results:

(1) It is unclear why 10% sucrose solution was used as a liquid instead of water, given that the study is focusing on the solid food source.

One motivation for using sucrose rather than water alone was to create a highly palatable environment and to test whether mice would prefer palatable liquid sucrose over HFD. However, the choice of liquid stimulus will ultimately depend on the end user and the specific experimental conditions of each lab implementing the Crunchometer. Future versions of the apparatus could also incorporate multiple sippers to deliver several tastants alongside solid food.

(2) It is unclear how essential the human verification is in the pipeline - results for Figure 1 keep referring to the verification as essential. Is that dispensable once the ML algorithms have been trained?

Human validation, also referred to as a human-in-the-loop approach, is a deliberate design feature of the Crunchometer rather than a limitation (also see answer to Reviewer 2). The outputs of machine-learning algorithms, no matter how accurate, require expert corroboration to confirm or reject the specific behaviors under study, particularly when the behavioral repertoire is as heterogeneous as feeding (which encompasses sniffing, gnawing, biting, hoarding, and manipulating the food item). For this reason, we view human oversight as a safeguard for scientific rigor that remains valuable even as more advanced algorithms (e.g., deep learning and convolutional neural networks) are incorporated into future versions of the pipeline. As noted above, we have implemented a graphical user interface (GUI) that enables batch sorting and rapid inspection of multiple snippets (using a photographic montage view strategy), substantially reducing manual curation time.

(3) The ability to extrapolate food quantity consumed is limited, with high variability. This limitation does not undercut the utility of the crunchometer, but should be highlighted as one of the parameters that are not suitable for this system. This limitation should be added to the limitations section.

We thank the reviewer for this constructive observation. We fully agree that, although the Crunchometer reliably detects feeding events and their temporal microstructure (bouts, meals, and latencies), extrapolating absolute food quantity consumed from acoustic signals is indirect and carries substantial variability and should not be the primary readout for studies that require precise gravimetric measurements. As recommended, we have now explicitly listed this limitation in the Limitations section of the Discussion:

"While the Crunchometer provides accurate temporal detection of bites and feeding microstructure, the estimation of absolute food mass consumed from bite-related acoustic signals shows considerable variability across trials and subjects. This limitation arises from individual differences in gnawing patterns, food fragmentation, and hoarding behavior. Accordingly, the Crunchometer is best suited for analyses of feeding dynamics and behavioral microstructure, whereas studies requiring precise quantification of ingested mass should complement the system with direct gravimetric measurements for example, real-time weighing of feeders."

(4) The ability to discriminate between gnawing and consummatory behavior is a strength (Figure 5), and these findings are important. However, it is unclear what can be made of mice that have 'gnawing' behavior in the fasted state (like in Figure 3). It seems they would need to be eliminated from the analysis with this tool?

We apologize for this misunderstanding. We have now more clearly indicated in Figure 3A that the cumulative feeding time reflects only Chow and HFD feeding bouts, excluding gnawing.

We now state: “The lower panel shows the cumulative feeding time (only for Chow and HFD pellets, gnawing is excluded) over a two-hour session for the fed (green) and fasted (purple) groups (n = 6 mice).”

Under normal physiological conditions, gnawing is an infrequent behavior in rodents. In our study, however, its frequency increased in the fasted state a change possibly attributable to heightened stress. This behavior was further exacerbated by chemogenetic manipulation, driving it to non-physiological levels.

(5) Why is there a post-semaglutide fed group and not a fasted group in Figure 4? It seems both would have been interesting, as one could expect an effect on feeding even 24h after semaglutide treatment. This would help parse the preference better because the animals eat such a small amount of semaglutide, that it is hard to compare to the fasted condition with saline treatment.

We thank the reviewer for this insightful suggestion. It would have been interesting to include a fasted post-semaglutide group, as it could provide relevant information about the lasting effect of an acute administration of semaglutide. However, we decided not to include this additional experimental condition because the semaglutide fasted mice displayed a markedly reduced food intake during the experimental session. An additional post-semaglutide fasted session would have required a prolonged food restriction (at least 24 hours), which we consider an unnecessarily stressful condition for the mice. Therefore, we decided to feed the mice once the experiment was completed. Nevertheless, we believe that comparing the food intake (grams) between the fed group shown in Figure 3C and the post-semaglutide fed group reported in Figure 4D provides insight into the lasting effect of semaglutide. The comparison reveals a remarkable reduction of food intake in the post-sem fed mice relative to the fed group, suggesting that the acute administration of semaglutide suppresses the feeding behavior for up to 24 hours.

(6) The identification of 'meal-related' neurons in the LH is another strength of the manuscript. Although there is currently insufficient data, could similar recordings be used to give a neurophysiological definition of a 'meal' duration/size? Typically, these were somewhat arbitrarily defined behaviorally. Having a neural correlate to a 'meal' would be a powerful tool for understanding how meals are involved in overall caloric intake.

We thank the reviewer for this insightful suggestion. We agree that the traditional behavioral criteria for defining meals, typically derived from log-survivor analyses of inter-pellet or inter-lick intervals, are operationally useful but ultimately arbitrary, and that a neurophysiologically grounded definition would be a valuable complement for the field.

Our current dataset was not designed to formally establish such a definition, and we want to be cautious about the logic of the problem: validating a neural criterion solely against the behavioral one it would replace is circular. A genuinely neural definition of a meal would need to be anchored to independent criteria, for example, its ability to predict the latency and size of the subsequent meal, its correspondence with post-prandial satiety markers, or its response to anorectic agents such as GLP-1 receptor agonists. This is a methodologically nontrivial undertaking that we believe deserves a dedicated follow-up study.

As preliminary evidence that such a problem is tractable, we note that the meal-related LH neurons identified here display sustained activity with onset and offset dynamics that broadly parallel the behaviorally defined meal boundaries (Figure 6), suggesting that meal structure is reliably encoded at the population level. A related approach, using neural activity to segment ingestive behavior at finer temporal scales, has been successful in our previous work on licking microstructure in the nucleus accumbens (Tellez, et al. 2012), and we consider the present findings a natural extension of that line of research to the larger meal timescale.

(7) The conclusion in the title of Figure 8 is premature, given the pilot nature and small number of neurons and mice sampled.

We appreciate this comment and agree with the reviewer. Accordingly, we have performed additional experiments on the Vglut2 glutamatergic population, in some cases using three-plane recordings, which substantially increased the yield to 386 glutamatergic neurons. As the reviewer anticipated, we observed a broad diversity of response profiles in this population, including neurons selective for liquid licking, for solid food intake, and for both food types. We also formally quantified these responses using ROC analysis, applying the same procedure to the Vgat GABAergic neurons (n = 79). These new findings have been incorporated into the revised manuscript (Results and Discussion). We thank the reviewer for prompting this extension of the analysis (see Manuscript).

Conclusion:

Overall, this report on the Crunchometer is well done and provides a valuable tool for all who study food intake and the behaviors around food intake. Clarification or answers to the points above will only further the utility and understanding of the tool for the research community. I am excited to see the future utility of this tool in emerging research.

We sincerely thank the Reviewer for these kind and encouraging words, and for the constructive feedback provided throughout the review. The clarifications and additional analyses prompted by these comments have substantially improved the manuscript, and we share the Reviewer's enthusiasm about the potential of the Crunchometer to contribute to future research on feeding behavior.

**Recommendations for the authors:**

**Reviewer #2 (Recommendations for the authors):**

(1) The authors have done a phenomenal job with the Introduction, highlighting the need for this tool, citing the history of feeding measurement systems and their relative strengths and weaknesses.

Thank you for your comment; we greatly appreciate your positive feedback.

(2) A limitation of Automated Pellet Dispensers is the possibility that the animals fail to consume the pellet after it has been retrieved from and registered by the device, potentially constraining accuracy.

We address this issue in the Introduction, specifically, we wrote:

“Current methods to monitor feeding behavior could be classified into four different classes…3 Automated Pellet Dispensers: Often integrated into operant conditioning chambers, these devices provide a controlled way of delivering food pellets. While devices like the open-source Feeding Experimentation Device (FED3) (Ali and Kravitz, 2018; Matikainen-Ankney et al., 2021), a pellet dispenser, are useful for measuring reinforcement, they alter the natural feeding patterns of mice, for example, requiring a simple action, such as a nose-poke can reduce overeating and weight gain in mice (Barrett et al., 2025). A further limitation is that FED3 may overestimate consumption if an animal retrieves and registers a pellet without actually consuming it. A significant strength of this method is its ability to enable closed-loop optogenetic stimulation concurrent with neuronal recordings.”

(3) I really appreciate the data in Figure 2G, where they displayed the results of an "outlier" animal, as behavior is extremely variable, and it's useful to see how this system deals with the variability of the subjects. This is again highlighted by mouse number 5 in Figure 3A, which exhibited profound gnawing behavior.

We thank the reviewer for this positive comment. Our decision to include the outlier animal in Fig. 2G and to report the atypical gnawing behavior of mouse 5 in Fig. 3A reflects a deliberate commitment to documenting inter-individual variability, which we consider a core strength rather than a limitation of behavioral work. We believe that such cases are particularly informative for evaluating the robustness of automated monitoring systems under behavioral-lab conditions.

(4) It would be useful to know if the mice had prior exposure to HFD, as I found it surprising that many animals consumed the chow at all, sometimes completely ignoring the HFD (fasted mouse 3). I only ask because in our experience, mice with constant exposure to both HFD and chow predominantly, if not always, consume the HFD over chow. This could have something to do with the way the food substrates are presented in this chamber.

We thank the reviewer for this point. Mice in this experiment did receive prior exposure to both Chow and HFD during the habituation phase, with at least two 30-min sessions in the experimental chamber with both diets available (no video was collected at this stage). The Chow and HFD feeders were identical in geometry, position, and accessibility, so we do not consider either environmental novelty or spatial bias to be the main driver of the pattern. Rather, we interpret the strong chow preference of fasted mouse 3 as a case of residual neophobia toward the HFD pellet. Since performing these experiments, we have refined our habituation protocol: pre-exposing animals to a single HFD pellet in their home cage, a familiar and safe environment, prior to any chamber session, greatly mitigates HFD neophobia in our hands. Familiarity with the novel food in a safe context thus appears to be the critical factor, rather than the duration of exposure in the experimental chamber. We have added this refinement to the Methods as a recommendation for future users of the Crunchometer.

“Behavioral protocol. All mice were habituated to the Crunchometer for 2 days before the recording session. Each habituation session lasted 30 minutes, during which two food pellets were placed in the chamber: one standard Chow pellet (LabDiet 5008) and one highly palatable high-fat diet (HFD) pellet (Research Diet, D12451). As a practical note, we recommend allowing the HFD to equilibrate to room temperature before the experiment and pre-exposing mice to a single HFD pellet in their home cage to attenuate neophobia prior to testing.”

(5) The authors claim saline or semaglutide was administered immediately before the start of the behavioral experiment, but given the time it takes for this drug to blunt appetite, I was somewhat surprised it led to such a rapid decrease in both chow and HFD intake. Could the authors comment on this? How quickly do these animals experience the malaise associated with these drugs? Also, this dose seems to be on the very high side, so I imagine it's making the animals feel quite sick and is probably a big reason why the effects last so long into the post-sem measurements. Was bodyweight tracked across this treatment? I'm not so convinced that sema treatment led to a loss of strong HFD preference, as the chow intake was already very low to begin with, and as mentioned above, it looks like the drug just led to a cessation of all intake. I'd just tamp down this claim of preference switch. It clearly reduced intake of both substrates, it's just harder to detect for the chow because it was already so low to begin with.

Thank you for these comments. We agree with the Reviewer and have toned down the claim regarding a switch in HFD/chow preference. In the revised Results section, we now explicitly acknowledge that further characterization is needed using chronic semaglutide treatment. Specifically, we added the following sentence:

"Future studies should use the Crunchometer to characterize changes in HFD/chow preference during 24-h monitoring under chronic semaglutide treatment."

In addition, we administered a single subcutaneous dose of semaglutide at 30 nmol/kg (0.123 mg/kg), following the protocol described by Zhang et al. (2023). In their study, pharmacokinetic analyses showed that plasma concentrations, measured by an ELISA assay that immunoreacts with both growth differentiation factor 15 (GDF15) and the intact N-terminal region of glucagon-like peptide-1 (GLP-1), increased shortly after administration of the 30 nmol/kg dose in C57BL/6 mice. Peak plasma concentration (Cmax = 43.1 nmol/L) was reached at 6.7 hours (Tmax), and levels returned to baseline by 24 hours post-administration, indicating complete drug clearance. Although this dose is relatively high, it was intentionally selected to produce a robust acute response from a single administration, as our objective was to assess the drug’s effects within a short, 2-hour observational window. Under these conditions, we observed a rapid reduction in food intake immediately following the onset of Crunchometer recording. While we do not exclude the possibility that these effects could be more pronounced over longer observation periods or with chronic dosing regimens, our study was strictly limited to a single acute exposure.

Although semaglutide is known to suppress food intake through multiple mechanisms, including stress and malaise measured by Conditioned Taste Aversion and release of stress hormones (Teixidor-Deulofeu et al., 2025), we do not believe that discomfort or malaise played a significant role in our study. While the mice did reduce their food intake during semaglutide administration, this reduction persisted for at least 24 hours after the final dose—at which point the drug was no longer present—suggesting a satiety-driven effect rather than one mediated by aversion. In this sense, previous studies have demonstrated that semaglutide continues to suppress food intake even when the aversive pathway mediated by Area Postrema GLP1R neurons is inhibited. Although blocking this pathway reduces flavour aversion, the anorexic effect remains, indicating that suppression of intake can be driven by satiety independently of nausea or malaise (Huang et al., 2024). In summary, although we selected a relatively high dose to ensure a detectable acute effect within our experimental window, this choice was grounded in previously published data, and our findings are consistent with established mechanisms of action for semaglutide.

Additionally, body weight data have now been included in Figure 4D. We observed a similar body weight loss of approximately 5% on the first day of drug administration, consistent with the findings reported by Zhang et al. (2023).

(6) The authors demonstrate that CNO administration prompted significant increase in liquid sugar intake in the last panel of Figure 5F as a confirmation that LH GABAergic neurons are implicated in processing reward, however given the above results it seems likely that these mice will drink anything including water (when not thirsty, thus in a non-rewarding scenario) or possibly aversive agents like quinine.

This is an interesting question, and we agree with the Reviewer. The original discovery by Jennings and Stuber showed that optogenetic activation of these GABAergic neurons induces voracious feeding and that Vgat mice kept licking for liquid rewards in an appetitive task (Jennings et al., 2015). We also acknowledge that prior work has shown LH GABAergic neuron activation can drive consumption of non-caloric and biologically irrelevant stimuli, including wood gnawing, water, or saccharin (Navarro et al., 2016). However, several lines of evidence support a role in reward/palatability processing rather than purely indiscriminate consumption. Our own lab (Garcia et al., 2021) showed that activation of LH Vgat+ neurons increased quinine intake only during water deprivation; in sated animals, activation failed to promote quinine intake. Instead, these neurons promoted overconsumption of sucrose when available, leading us to conclude that LH Vgat+ neurons increase the drive to consume the nearest food, but this drive is potentiated by the palatability of the tastant. In non-human primates, LH GABA activation drives goal-directed eating predominantly for palatable food (Ha et al., 2024), supporting a reward-related function across species. Together, these findings indicate that while LH GABAergic activation does broadly promote consumption, the selectivity toward palatable stimuli observed in Figure 5F is consistent with a reward-related function.
